# Editorial: Antibody-mediated thrombotic disease

**DOI:** 10.3389/fimmu.2026.1800389

**Published:** 2026-02-12

**Authors:** Konstantine Halkidis, Szumam Liu, Shruti Chaturvedi

**Affiliations:** 1 Division of Hematologic Malignancies and Cellular Therapeutics, Department of Internal Medicine, Institute for Reproductive and Developmental Sciences, University of Kansas Medical Center, Kansas City, KS, United States; 2 Department of Pathology and Laboratory Medicine, Institute for Reproductive and Developmental Sciences, University of Kansas Medical Center, Kansas City, KS, United States; 3 Division of Hematology, Department of Medicine, Johns Hopkins University, Baltimore, MD, United States

**Keywords:** antiphospholid syndrome, autoimmune disease, complement mediated diseases, PF4 antibodies, thrombosis

Thrombosis represents one of the most devastating consequences of many autoimmune diseases. Indeed, almost all patients with an autoantibody-mediated disorder are at increased risk of thrombosis, either as a direct consequence of the mechanistic effects of pathogenic antibodies or indirectly because of a general pro-inflammatory state. New insights are needed to help mitigate this risk. In this Research Topic, our contributors provide a state-of-the-art overview of the current state of knowledge in antiphospholipid syndrome, complement-mediated hemolytic uremic syndrome, and anti-PF4 related immunothrombosis.

Several of the articles in our Research Topic provide fresh insights on how elevated levels of circulating antibodies in the plasma of patients with antiphospholipid syndrome (APS) may correlate with disease manifestations related to thrombosis. Niznik et al. describe a new classification category for patients with APS at increased risk of recurrent thrombosis despite optimal anticoagulation: “Terrible APS”, or TrAPS. Villani et al. report their findings on antibodies that bind to a complex of β2-glycoprotein I (β2-GPI) and platelet factor 4 (PF4) being enriched in patients with thrombotic APS; elevated levels of these anti-β2-GPI/PF4 antibodies also correlated with venous complications and appear to stimulate platelet activity. Cabrera-Marante et al. explore the potential utility of antiphospholipid antibodies not currently used as part of the diagnostic criteria for the disease, and found that increased levels of anti-phosphatidylserine/prothrombin (aPS/PT) IgG and IgM, as well as anti- β2-GP1 IgA, are associated with thrombotic and obstetric manifestations of APS. García-Camarero et al. investigated the extent to which the presence of elevated levels of antiphospholipid and antinuclear antibodies correlate with outcomes in coronary artery disease (CAD) progression, finding that CAD patients more frequently exhibited positive anti-cardiolipin antibodies compared with controls, but no significant association amongst the antibody panel they explored with outcomes in CAD. Li et al. performed a systemic review and meta-analysis to explore the potential of antibodies that specifically recognize domain 1 on β2-GP1 (anti-β2-GP1-D1) in clinical management of APS patients; overall, anti-β2-GP1-D1 antibodies can distinguish patients with APS from those with other autoimmune diseases and healthy controls, but may not be as effective at differentiating APS from an antiphospholipid antibody carrier state.

In addition to exploring antibodies as prognostic and/or predictive tools in APS, we have intriguing contributions that illuminate insights into antibody-mediated thrombotic disease pathophysiology. Yang et al. provide a comprehensive and engaging review of mechanisms of antiphospholipid antibody-mediated thrombosis in APS, a complex disease with myriad end-organ manifestations and effects on hemostasis. Álvarez et al. utilized human umbilical vein endothelial cells (HUVECs) exposed to IgG from patients with vascular and obstetric APS or IgG purified from healthy women with “proven gestational success”. The IgG from APS patients appeared to bind to the endothelial surface more than control and also enhanced platelet-rich plasma-dependent procoagulant activity on the endothelial surface in a statistically significant manner. Müller et al. summarize advances in our understanding of anti-PF4 related immunothrombosis, reviewing the pathophysiologic role of anti-PF4 antibodies in disorders such as heparin-induced thrombocytopenia (HIT), vaccine-induced immune thrombocytopenia and thrombosis (VITT), and monoclonal gammopathies of thrombotic significance (MGTS), and how the Fcγ receptor IIA (FcVRIIA) on platelets ties them all together. Alyamany et al. describe evolving insights into the pathophysiology, diagnosis, and treatment of complement-mediated hemolytic uremic syndrome (CM-HUS) in their stimulating review.

Antibody-mediated thrombotic disease remains one of the frontiers of medicine. Though broad in scope and scale, this Research Topic covers but a fraction of the field, which also includes immune thrombotic thrombocytopenic purpura (1). Coupled with the new ACR/EULAR criteria for APS, these studies will help usher in a new era in the care of APS patients (2). Our knowledge of the role of PF4 in thrombosis is still expanding and will hopefully manifest in novel diagnostic and therapeutic tools. Deeper insights into CM-HUS are needed to improve the lives of patients suffering from the disease. As is clear from this Research Topic, recent advances provide tremendous insight into these disorders, but there is much work to be done to help translate these findings into clinical practice.
